# Identification of molecular markers of bipolar cells in the murine retina

**DOI:** 10.1002/cne.21639

**Published:** 2008-04-10

**Authors:** Douglas S Kim, Sarah E Ross, Jeffrey M Trimarchi, John Aach, Michael E Greenberg, Constance L Cepko

**Affiliations:** 1 Department of Genetics, Harvard Medical SchoolBoston, Massachusetts 02115; 2 Neurobiology Program, Department of Neurology, Children's HospitalBoston, Massachusetts 02115; 3 Howard Hughes Medical Institute, Harvard Medical SchoolBoston, Massachusetts 02115

**Keywords:** retina, bipolar cells, *Bhlhb4*, gene expression, microarray, mouse

## Abstract

Retinal bipolar neurons serve as relay interneurons that connect rod and cone photoreceptor cells to amacrine and ganglion cells. They exhibit diverse morphologies essential for correct routing of photoreceptor cell signals to specific postsynaptic amacrine and ganglion cells. The development and physiology of these interneurons have not been completely defined molecularly. Despite previous identification of genes expressed in several bipolar cell subtypes, molecules that mark each bipolar cell type still await discovery. In this report, novel genetic markers of murine bipolar cells were found. Candidates were initially generated by using microarray analysis of single bipolar cells and mining of retinal serial analysis of gene expression (SAGE) data. These candidates were subsequently tested for expression in bipolar cells by RNA in situ hybridization. Ten new molecular markers were identified, five of which are highly enriched in their expression in bipolar cells within the adult retina. Double-labeling experiments using probes for previously characterized subsets of bipolar cells were performed to identify the subtypes of bipolar cells that express the novel markers. Additionally, the expression of bipolar cell genes was analyzed in *Bhlhb4* knockout retinas, in which rod bipolar cells degenerate postnatally, to delineate further the identity of bipolar cells in which novel markers are found. From the analysis of *Bhlhb4* mutant retinas, cone bipolar cell gene expression appears to be relatively unaffected by the degeneration of rod bipolar cells. Identification of molecular markers for the various subtypes of bipolar cells will lead to greater insights into the development and function of these diverse interneurons.

Bipolar cells in the vertebrate retina are the first relay inteneurons in the visual system, connecting rod and cone photoreceptor cells to amacrine and ganglion cells. They are critical for routing and processing of photoreceptor cell signals sent to specific postsynaptic targets. Bipolar cells assume diverse but specific morphologies, evident in their varied axon lengths, terminal field widths, and cell body positions in the inner nuclear layer (INL). For example, bipolar cells postsynaptic to rod photoreceptor cells have cell bodies positioned closest to the outer plexiform layer (OPL), extend axons to the deepest part of the inner plexiform layer (IPL), and form synapses with specific amacrine cells. Cone bipolar cells have cell bodies both close to and relatively distant from the OPL and ramify axonal processes throughout the IPL.

In addition to morphological diversity, molecular differences are found in gene and protein expression among bipolar neurons. Correlation of antibody staining patterns with morphologies of distinct bipolar cell subsets has led to definition of 10 bipolar cell subtypes in the rodent retina (Haverkamp et al., 2003a; Ghosh et al., 2004; Pignatelli and Strettoi, 2004). Characterization of gene expression patterns has aided in elucidating roles of specific bipolar cell subtypes in circuits critical for different aspects of vision (Vardi and Morigiwa, 1997; Vardi, 1998). Additionally, investigators studying retinogenesis have utilized a limited set of bipolar cell molecular markers to describe the development of normal and genetically altered retinas (Bramblett et al., 2004; Chow et al., 2004; Ohtoshi et al., 2004; Cheng et al., 2005; Wan et al., 2005). Identification of a more complete set of markers could lead to further insights into retinal physiology and development.

Investigation of genes differentially expressed among bipolar cells could enhance understanding of their function and ontogeny. Included among known molecules expressed in different subtypes are neurotransmitter receptors, intracellular signal transduction components, calcium-binding proteins, and transcription factors (Greferath et al., 1990; Berrebi et al., 1991; Euler and Wässle, 1995; Burmeister et al., 1996; Takebayashi et al., 1997; Vardi and Morigiwa, 1997; Koulen et al., 1998; Fletcher et al., 1998; Vardi, 1998; Baas et al., 2000; Haeseleer et al., 2000; Chow et al., 2001; Ohtoshi et al., 2001; Haverkamp et al., 2003a,b; Huang et al., 2003; Ghosh et al., 2004; Blackshaw et al., 2004). One transcription factor, basic helix-loop-helix transcription factor b4 (Bhlhb4), is specifically expressed in rod bipolar cells (Bramblett et al., 2004). Members of the bHLH class of transcription factors are critical for development and maintenance of retinal cells and neural cells generally (Ross et al., 2003, Pennesi et al., 2003; Hatakeyama and Kageyama, 2004). Analysis of *Bhlhb4* knockout mice has revealed that Bhlhb4 is required for rod bipolar cell survival in the late postnatal period after differentiation has occurred and during adulthood (Bramblett et al., 2004).

In this study, we explored the molecular diversity of bipolar cells by identifying novel genetic markers of these neurons through the use of genomic expression screening methods and RNA in situ hybridization. Retinal gene expression data from a combination of microarray and serial analysis of gene expression (SAGE) studies led to identification of candidate bipolar cell genes. Candidates were then evaluated for enriched expression in bipolar cells by using RNA in situ hybridization. Ten novel molecular markers enriched to varying degrees in their expression in bipolar cells were found. The specific bipolar cell subtypes expressing validated bipolar cell genes were assessed with double-labeling by using previously characterized bipolar cell markers. Additionally, newly identified bipolar cell markers were used to analyze *Bhlhb4* knockout retinas. The results reveal complex patterns of gene expression, suggesting that overlapping combinations of transcription factors likely control bipolar cell identity and function.

## MATERIALS AND METHODS

### In vitro electroporation

The previously characterized 4.7-kb calcium-binding protein 5 (*Cabp5*) promoter (Matsuda and Cepko, 2004) was cloned upstream of a green fluorescent protein (GFP; Clontech, Mountain View, CA) sequence. CD-1 mouse pups (Charles River Laboratories, Wilmington, MA) were sacrificed at postnatal day (P)0, and retinas were dissected and electroporated as described (Matsuda and Cepko, 2004). Retinas were cultured for 8 days on floating Nuclepore Track-Etch membranes (Whatman, Florham Park, NJ) in 10% fetal bovine serum (Invitrogen, Carlsbad, CA), 45% Dulbecco's modified Eagle's medium (DMEM; Invitrogen), 45% F12 nutrient mixture (Invitrogen), 100 U/mL penicillin (Invitrogen), 100 mg/mL streptomycin (Invitrogen), and 2 mM L-glutamine (Sigma, St. Louis, MO). All mice were used in accordance with the guidelines for animal care and experimentation established by the National Institutes of Health and the Harvard Medical Area Standing Committee on Animals.

### Gene microarray analysis

Freshly dissected or transfected retinas were dissociated by papain digestion, and single cells were picked from a tissue culture dish by using a fine glass pipette and an inverted microscope as in Trimarchi et al. (2007). Previous estimates suggest that picogram quantities of RNA are found per cell (Iscove et al., 2002). cDNA from single cells was prepared by using an oligo-dT-containing primer and amplified by a 35-cycle reverse transcriptase-polymerase chain reaction (RT-PCR) protocol detailed in Trimarchi et al. (2007), which was based on previous single-cell PCR amplification methods (Brady and Iscove, 1993; Dulac and Axel, 1995; Tietjen et al., 2003). cDNA quality was assessed as an indirect measure of RNA quality after PCR by gel electrophoresis, and cDNA with strong bands ranging from approximately 500 to 2,000 bp was used. Previous investigators have shown that this single-cell PCR strategy yields results that are well correlated with input RNA levels, that the cDNA is biased for the 3′ end of genes because an oligo-dT primer is used in the reverse transcription, and that as few as 10 copies of an RNA transcript can be reliably detected after RT-PCR by using microarrays (Tietjen et al., 2003). cDNA probes were hybridized to Affymetrix Mouse Genome 430 2.0 microarrays as in Trimarchi et al. (2007). Signals from microarrays were globally scaled with a target intensity set to 500 by using Affymetrix Microarray Suite software (MAS 5.0).

### SAGE data mining

The SAGE library data generated in Blackshaw et al. (2004) were used. Nearest neighbor analysis was conducted by selecting neighbor genes whose temporal expression patterns, based on reliably assigned gene tag counts, displayed the minimal Euclidean distances to the pattern of a known bipolar cell or rod photoreceptor cell gene. Prior to performing analysis, the temporal expression pattern for each gene was normalized by fixing the tag average and tag standard deviation to common values. This normalization facilitated relative comparison of genes whose absolute expression levels varied widely.

### RNA in situ hybridization and antibody staining

Retinas were dissected in phosphate-buffered saline (PBS; pH 7.4), fixed in 4% paraformaldehyde in PBS for 30 minutes at 22°C, rinsed three times, and cryoprotected for 1 hour in 30% sucrose in PBS. Retinas were embedded in OCT (Sakura Finetek, Torrance, CA), and 20-μm sections were cut and slide-mounted by using a cryostat microtome. Riboprobes were polymerized, and RNA in situ hybridization was performed as in Murtaugh et al. (1999) with modifications detailed in Trimarchi et al. (2007). Table 1 shows a list of the antisense riboprobes used and the Genbank accession numbers of sequences from which riboprobes were derived. As negative controls, sense probes were also hybridized separately to retinal sections.

**Table 1:** RNA In Situ Hybridization Probes

Gene name	Genbank accession no.	Unigene	Polymerase used for antisense
Basic helix-loop-helix transcription factor B4 (*Bhlhb4*)	NM_080641, bases 165-989	Mm.134062	T7
Carbonic anhydrase 8 (*Car8*)	AI838156	Mm.119320	T3
Carbonic anhydrase 10 (*Car10*)	NM_028296, bases 322-875	Mm.342160	T7
*C. elegans* ceh-10 homeodomain containing homolog (*Chx10*)	BF461223	Mm.4405	T3
Contactin 4 (*Cntn4*)	BE948217	Mm.321683	T3
Glutamate receptor, metabotropic 6 (*Grm6*)	BI732193	Mm.134265	T7
LIM homeobox gene 3 (*Lhx3*)	BE987423	Mm.15655	T7
Neurofascin (*Nfasc*)	BE986133	Mm.380307	T3
Og9 homeobox gene (*Og9x*)	BE950656	Mm.134360	T3
Paired box gene 6 (*Pax6*)	NM_013627, bases 395-2412	Mm.3608	T3
Purkinje cell protein 2 (*Pcp2*)	BE954628	Mm.41456	T3
Protein kinase c, alpha (*Prkca*)	BE949837	Mm.222178	T3
Secretagogin (*Scgn*)	BE987143	Mm.255667	T3
Transient receptor potential action channel subfamily M member 1 (*Trpm1*)	BE985968	Mm.38875	T7
RIKEN cDNA 2300002D11 gene (*2300002D11Rik*)	BE987910	Mm.151594	T3
RIKEN cDNA 6330514A18 gene (*6330514A18Rik*)	BE985967	Mm.17613	T3

For double-labeling, P14 retinas were dissected and dissociated by incubation in a solution containing 25 U/mL papain (Worthington, Lakewood, NJ), 3 mM L-cysteine, 0.5 mM EDTA, and 0.3 mM β-mercaptoethanol in Hanks' balanced salt solution (Invitrogen) at 37°C for 10 minutes. Digestion was stopped by addition of 1.5 volumes of DMEM containing 10% fetal bovine serum, and retinas were incubated in 50 U/mL DNaseI (Roche, Indianapolis, IN) at 37°C for 5 minutes. Following manual trituration with a pipettor, cells were rinsed once in DMEM containing 10% fetal bovine serum and then placed onto slides coated with poly-d-lysine (Sigma). Slides were processed, and double-label in situ hybridization was carried out as in Trimarchi et al. (2007). A subset of slides was singly labeled with a riboprobe and then stained with a rabbit polyclonal anti-calbindin D 28K (Calb) antibody (Sigma, C9848; 1:300) or a mouse monoclonal anti-glutamine ammonia ligase (Glul) antibody (Chemicon, Temecula, CA, MAB302; 1:500). The anti-Calb antibody was made from mice immunized with purified bovine kidney calbindin D 28K, and staining of adult retinal sections resulted in a pattern of immunoreactivity that was similar to previous observations (see Supplementary Fig. 3; Lee et al., 2006). The anti-Glul antibody was produced from mice immunized with glutamine ammonia ligase purified from sheep brain, and staining of adult retinal sections revealed a pattern of immunoreactivity that was similar to previous observations (see Supplementary Fig. 3; Rhee et al., 2007). Previous Western blot analysis showed that this antibody recognizes a single 45-kD protein in adult retinal tissue (Chang et al., 2007a).

Slides were washed twice in PBS/0.1% Triton X-100 (v/v) for 5 minutes at 22°C, blocked with 10% normal goat serum in PBS/0.1% Triton X-100 for 1 hour at 22°C, and incubated with primary antibody in 2% normal goat serum in PBS/0.1% Triton X-100 overnight at 4°C. Slides were washed three times in PBS for 10 minutes at 22°C, incubated with either a Cy2-conjugated goat anti-rabbit IgG or a Cy2-conjugated goat anti-mouse IgG secondary antibody (Jackson ImmunoResearch, West Grove, PA; 1:250) in 10% normal goat serum in PBS/0.1% Triton X-100 for 2 hours at 22°C, and rinsed three times in PBS for 10 minutes at 22°C. After double-labeling, cells were stained with 4′,6-diamidino-2-phenylindole (DAPI; 1 μg/mL in PBS, 22°C, 5 minutes; Roche).

Digital images of Cy3-tyramide (Perkin Elmer, Wellesley, MA), Alexa 488-tyramide (Invitrogen), Cy2-antibody, and DAPI-stained cells were processed to quantitate results by using custom image analysis software (J. Aach and G. Church, Harvard Medical School). DAPI-labeled nuclei were used to segment red/green/blue composite images into individual cell images. This was done by applying a particle size filter to remove labeled debris, blurring DAPI-labeled nuclei through averaging of small windows of pixels to smooth intensities, dividing nuclei from one another with borders by using a watershedding algorithm, and fitting ellipses around each nucleus inside of its border. Red and green pixel intensities inside of each segmented nucleus plus a small surrounding perinuclear region were quantitated. Thresholds were set for mean signal intensities for scoring positive cells, and negative, single-positive, and double-positive cells were counted. A subset of images was counted independently to verify accuracy of the automated analysis. Representative photomicrographs are presented as red/green/blue (RGB) images merged in Adobe Photoshop software (Adobe Systems, San Jose, CA), through which raw RGB intensity levels were adjusted visually and uniformly across photographs from the same hybridization to reflect thresholds set for quantitation.

### Gene targeting

An approximately 5-kb *Hin*dIII/*Not*I DNA fragment encompassing the *Bhlhb4* gene was isolated by PCR from 129/Sv genomic DNA. A loxP-flanked *PGK*-neomycin resistance cassette in reverse orientation was introduced into the *Kpn*I site approximately 1 kb upstream of the *Bhlhb4* gene. A third loxP sequence was introduced approximately 300 bases distal to the *Bhlhb4* 3′ UTR, between adjacent *Bsr*GI and *Mun*I restriction sites. A *PGK*-diptheria toxin gene cassette was introduced at the 3′ end of the *Bhlhb4* locus. This vector was electroporated into mouse 129 J1 embryonic stem (ES) cells and selected as described (Li et al., 1992). Clones were screened by Southern blot analysis. Eight of 96 clones were correctly targeted. A null allele was generated by transiently transfecting targeted ES cells with a *Cre* recombinase plasmid, pOG231 (from S. O'Gorman, Case Western Reserve University). Targeted ES cell clones harboring a deletion of both *Bhlhb4* and *PGK*-neomycin were identified with PCR by using primers that flanked the outermost loxP sites (*Bhlhb4* null allele primers; described below).

ES cells carrying the deletion allele were microinjected into C57BL/6 blastocysts, and chimeras were tested for germline transmission of the mutant allele by breeding to C57BL/6 females and PCR genotyping of pups. Primers used for genotyping were as follows: wild-type allele primers: 5′-AGCTCAAGTCGCTGTCGGG-3′, 5′-TCGAAGGCTTCGTCCTCGTC-3′; *Bhlhb4* null allele primers: 5′-CGACCTCTTGCTGAAACCACAG-3′, 5′-GCCGTAGAAGGATTCCAAACCAG-3′. Wild-type (WT) and mutant mice maintained on a mixed 129/Sv X C57BL/6 background were used.

## RESULTS

### Gene expression screening using single retinal cells

In an initial effort to identify candidate novel molecular markers enriched in bipolar cells, oligonucleotide microarrays were used to characterize gene expression in single bipolar cells from the mouse retina. Candidate genes identified in this manner were subsequently validated as being enriched in bipolar cells by RNA in situ hybridization (see further below). Because bipolar cells comprise only a small fraction of total retinal cells (∼10%) compared with rod photoreceptor cells (>70%; Young, 1985), individual bipolar cells, instead of whole retinas, were utilized to enrich for genes of interest. Single bipolar cells were picked from enzymatically dissociated mouse retinas based on expression of a GFP reporter construct driven by the *Cabp5* gene promoter transfected into retinas at P0 and harvested after 8 days of culture. This *Cabp5* promoter has been shown previously to be active in rod bipolar cells and a limited set of cone bipolar cells (Matsuda and Cepko, 2004).

By using a previously described, sensitive RT-PCR-based strategy (Trimarchi et al., 2007; see also Materials and Methods), cDNA from four individual bipolar cells was amplified and then hybridized to microarrays (Table 2; Cabp5 Bipolar A3, A4, B1, and B2). To generate additional expression profiles for comparison, randomly chosen single cells from freshly dissected retinas were picked at P5 and from adults and identified retrospectively as bipolar cells (Table 2; P5 Bipolar A3), rod photoreceptor cells (Adult Rod 1 and 2), or Müller glial cells (Adult Müller Glia 1 and 15) based on expression of known markers. For bipolar cells, these known genes included *Cabp5* (Haeseleer et al., 2000) and *C. elegans ceh-10* homeodomain-containing homolog (*Chx10*; Liu et al., 1994; Burmeister et al., 1996). For rod photoreceptor cells, these previously characterized molecular markers were cGMP-specific phosphodiesterase 6b (*Pde6b*; Hurwitz et al., 1985; Baehr et al., 1991; Chang et al., 2007b), cyclic nucleotide gated channel α1 (*Cnga1*; Kaupp et al., 1989), and rhodopsin (*Rho*; Jan and Revel, 1974; Molday and MacKenzie, 1983). For Müller glial cells, these known genes included retinaldehyde binding protein 1 (*Rlbp1*; Bunt-Milam and Saari, 1983) and glutamate-ammonia ligase (*Glul*; Riepe and Norenburg, 1977).

**Table 2:** Scaled Fluorescence Signal Intensity Levels from Microarrays of Single Mouse Retinal Cells^1^

			Fluorescence signal intensities										
(a)	(b)	(c)	(d)	(e)	(f)	(g)	(h)	(i)	(j)	(k)	(l)	(m)	(n)
Probe set ID	Unigene	Gene name	P5 bipolar A3	Cabp5 bipolar A3	Cabp5 bipolar A4	Cabp5 bipolar B1	Cabp5 bipolar B2	Adult rod 1	Adult rod 2	Adult Müller glia 1	Adult Müller glia 15	Ratio of signal intensities of bipolar cells to rod photoreceptor + Müller glial cells	*P* value
1451826_at	Mm.103669	Calcium binding protein 5 (*Cabp5*)	7,874	21,964	18,135	40,043	37,690	395	23	155	73	155.6	0.0042
1424944_at	Mm.41456	Purkinje cell protein 2 (*Pcp2*)	13	7,342	4,364	259	4,527	41	21	224	12	44.3	0.0406
1450945_at	Mm.222178	Protein kinase C α (*Prkca*)	143	3,273	7,272	9,010	2,643	28	200	184	26	40.9	0.0243
1419628_at	Mm.4405	*C. elegans* ceh-10 homeodomain-containing homolog (*Chx10*)	4,718	24,762	22,820	53,259	73,768	168	24	12	18,707	7.6	0.0343
1438782_at	Mm.321683	Contactin 4 (*Cntn4*)	3	12,582	6,377	26,568	23,591	527	141	14	4	80.6	0.0240
1427482_a_at	Mm.119320	Carbonic anhydrase 8 (*Car8*)	96	6,773	18,800	24,431	25,708	620	48	244	50	63.0	0.0174
1453008_at	Mm.151594	RIKEN cDNA 2300002D11 gene (*2300002D11Rik*)	14,214	25,518	23,537	16,469	13,327	1,082	60	386	54	47.0	0.0002
1457946_at	Mm.134360	Og9 homeobox gene (*Og9x*)	191	6,663	9,945	17,860	18334	168	634	190	40	41.0	0.0165
1424547_at	Mm.342160	Carbonic anhydrase 10 (*Car10*)	106	915	1,109	3,104	658	111	33	24	66	20.2	0.0471
1436205_at	Mm.380307	Neurofascin (*Nfasc*)	221	8,476	9,099	2,554	9,313	858	302	211	12	17.2	0.0180
1425041_at	Mm.15655	LIM homeobox protein 3 (*Lhx3*)	10,281	56	42	71	7	411	93	386	116	8.3	0.2271
1419740_at	Mm.1372	Phosphodiesterase 6B, cGMP, rod receptor, β polypeptide (*Pde6b*)	21	7,981	20	38	193	514,284	167,133	28	22	0.010	0.0786
1451763_at	Mm.23793	Cyclic nucleotide gated channel α1 (*Cnga1*)	22	1,979	7	14	15	194,172	159,622	28	2,985	0.005	0.0443
1451617_at	Mm.2965	Rhodopsin (*Rho*)	5	955	9	26	3,523	710,723	272,295	12,406	16	0.004	0.0667
1418310_a_at	Mm.41653	Retinaldehyde binding protein 1 (*Rlbp1*)	3	4,521	26	28	13,236	34	7	168,251	109,338	0.051	0.0586
1426236_a_at	Mm.210745	Glutamate-ammonia ligase (*Glul*)	41	3,798	4	13	282	434	296	221,720	89,714	0.011	0.0684

1 Signals are shown from a P5 bipolar cell (column d), four bipolar cells transfected with a *Cabp5:GFP* reporter construct at P0 and picked from retinas after 8 days in culture (columns e–h), two adult rod photoreceptor cells (columns i–j), and two adult Müller glial cells (columns k,l). For each gene, the ratio of the average signal of bipolar cells to the average signal of all other cells is shown (column m). The average bipolar signal was compared with the average signal of all other cells by one-tailed Student's t-test, and resulting *P* values are shown (column n). Candidate and known bipolar cell-enriched genes had ratios greater than 7.6, whereas known rod photoreceptor and Müller glial cell-enriched genes had ratios less than 0.051.

The fluorescence signal intensity levels from the microarrays of five bipolar cells were compared with those from two rod photoreceptor cells and two Müller glial cells. This comparison was done to enrich for genes expressed relatively specifically in bipolar cells and to filter out more widely expressed genes in at least some other retinal cell types. Table 2 shows the intensity levels for selected gene sequences from the single cells. The signal intensities for several previously characterized bipolar cell genes, including *Cabp5*, Purkinje cell protein 2 (*Pcp2*; Berrebi et al., 1991), protein kinase C α (*Prkca*; Greferath et al., 1990), and *Chx10*, were enriched between 8- and 156-fold when the average signal intensities for bipolar cells were compared with those for rod photoreceptor and Müller glial cells, confirming the utility of this method in identifying genes enriched in their expression in bipolar cells.

For each of these genes, the average signal intensity for the bipolar cells was significantly greater than the average signal intensity for the rod photoreceptor and Müller glial cells (*P* < 0.05, one-tailed Student's t-test, Table 2). Table 2 also includes signal intensities for several selected genes that were significantly enriched in the single bipolar cells and whose retinal expression has not been previously characterized. These genes, which exhibited some of the highest signal intensity ratios when comparing bipolar cells with other cells in the filtered results, were of particular interest because the possibility that they could be new bipolar cell-enriched molecular markers was substantial given that known bipolar cell genes were similarly enriched. The novel candidate bipolar cell genes were enriched between 8- and 81-fold and included contactin 4 (*Cntn4*), carbonic anhydrase 8 (*Car8*), RIKEN cDNA 2300002D11 gene (*2300002D11Rik*), Og9 homeobox gene (*Og9x*), carbonic anhydrase 10 (*Car10*), and neurofascin (*Nfasc*). For each of these genes, the average signal intensity for the bipolar cells was also significantly greater than the average signal intensity for the rod photoreceptor and Müller glial cells (*P* < 0.05, one-tailed Student's t-test, Table 2). For comparison, signal intensities for a subset of known rod photoreceptor (*Pde6b*, *Cnga1*, *Rho*) and Müller glial cell genes (*Rlbp1*, *Glul*) are shown in Table 2.

As expected for these rod photoreceptor and Müller glial cell genes, the signal intensity ratios for the bipolar cells to the rod photoreceptor and Müller glial cells were low, ranging from 0.004 to 0.051. These six candidate bipolar cell genes were demonstrated to be expressed in an enriched manner in bipolar cells, as assessed by RNA in situ hybridization (see further below). This limited set of single cells, while insufficient to provide the statistical power for identification of all bipolar cell-enriched genes, or to compare different cell types quantitatively, was sufficient for the purpose of screening for candidate novel bipolar cell molecular markers. Other genes exhibited high signal intensities in single bipolar cells and low signal intensities in rod photoreceptor and Müller glial cells, but these genes were not expressed in an enriched manner in bipolar cells as assessed by RNA in situ hybridization and so were not pursued further (data not shown).

### Identification of bipolar cell candidate genes by using SAGE

In an additional effort to identify candidate novel bipolar cell molecular markers, retinal SAGE data were also examined (Blackshaw et al., 2004). A previous analysis of gene expression during mouse development was carried out with SAGE by using 10 retinal cDNA libraries generated at various stages between embryonic day (E)12.5 and adulthood. Bipolar cell-enriched molecular markers were previously identified based on large-scale RNA in situ hybridization studies of genes shown to have dynamic temporal expression patterns within the SAGE data (Blackshaw et al., 2004). To mine the SAGE data further for additional genes enriched in bipolar cell expression, a nearest neighbor method was applied. Because bipolar cells are born in a discrete developmental period and differentiate relatively late (Young, 1985), genes with late onset of expression might be novel bipolar cell genes. A search was carried out by using nearest neighbor analysis and known bipolar cell genes that have late onset of expression. Candidate genes with temporal gene expression patterns that displayed minimal Euclidean distances to patterns for known bipolar cell genes were selected for further analysis by RNA in situ hybridization (see further below).

**Fig. 1:**
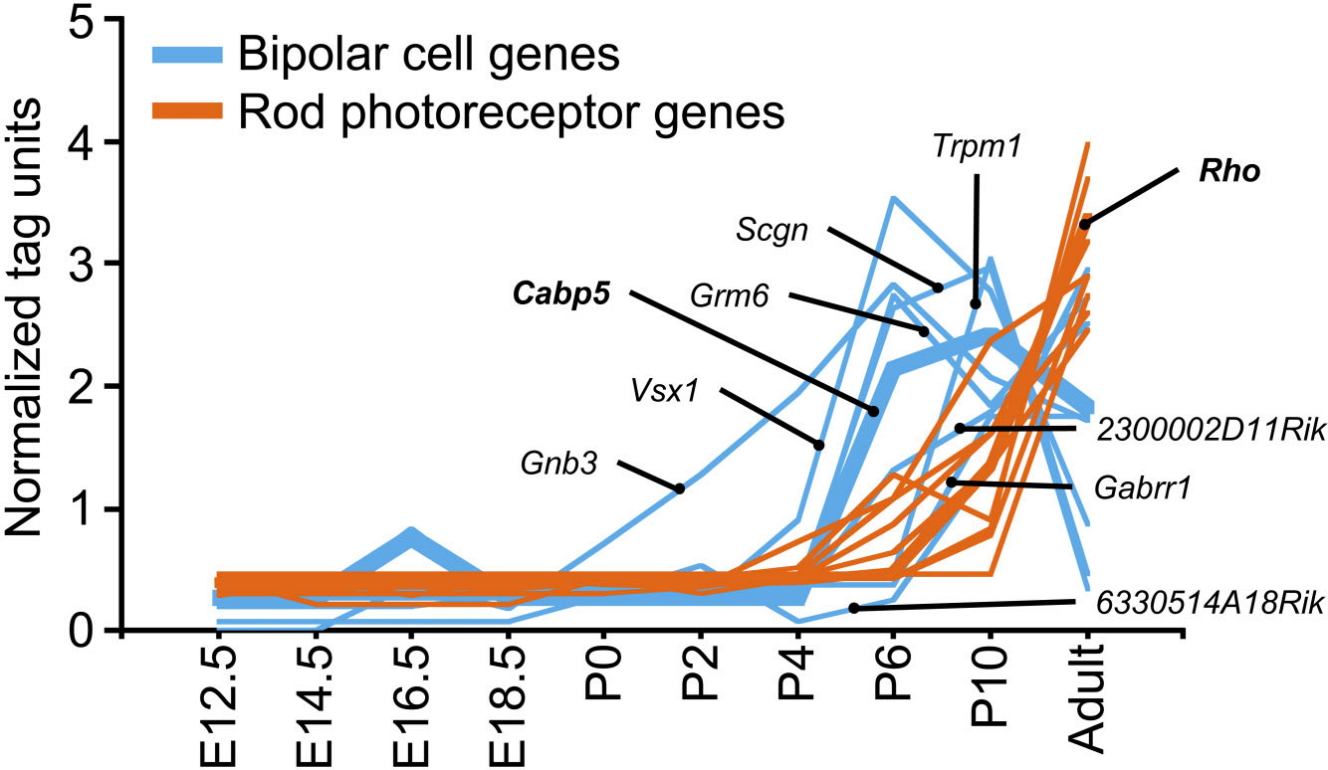
Temporal expression patterns of mouse bipolar cell and rod photoreceptor cell genes. Relative expression levels of selected genes are plotted as normalized SAGE tag units over developmental time between E12.5 and P10 and in adulthood. Patterns for *Cabp5* (thick blue line) and neighboring bipolar cell genes (thin blue lines; *Grm6*, *Gabrr1*, *Vsx1*, *Gnb3*, *2300002D11Rik*, *Scgn*, *Trpm1*, *6330514A18Rik*). Patterns for rhodopsin (thick orange line) and neighboring rod photoreceptor cell-enriched genes (thin orange lines; *Guca1a*, *Aipl1*, *Gnat1*, *Rom1*, *Gngt1*, *Guca1b*, *Grk1*, *Pde6g*) are shown.

The temporal expression patterns for the known bipolar cell gene *Cabp5* (Haeseleer et al., 2000) and eight genes with similar profiles are shown in Figure 1. The onset of expression for these genes was from P0 to P6.5, and the relative expression levels peaked between P6.5 and adulthood. This set included genes previously shown to be expressed in bipolar cells, such as metabotropic glutamate receptor 6 (*Grm6*; Nakajima et al., 1993; Vardi and Morigiwa, 1997), γ-aminobutyric acid (GABA)-C receptor rho subunit 1 (*Gabrr1*; Koulen et al., 1998), visual system homeobox 1 homolog (*Vsx1*; Chow et al., 2001,^2004^; Ohtoshi et al., 2001,^2004^), and G protein β3 (*Gnb3*; Huang et al., 2003), confirming the utility of this method in identifying genes enriched in their expression in bipolar cells. Also included was *2300002D11Rik*, a gene identified as a candidate bipolar cell molecular marker from the microarray screening.

Figure 1 also shows the temporal expression pattern of three additional genes with relatively late onset of expression: secretagogin (*Scgn*), transient receptor potential action channel subfamily M member 1 (*Trpm1*), and RIKEN cDNA 6330514A18Rik gene (*6330514A18Rik*). The Euclidean distance between the average temporal expression pattern of these eight known and candidate bipolar cell genes and the pattern of *Cabp5* was 4.01 ± 0.42 normalized tag units (average ± SEM). For comparison, this distance was significantly smaller than the distance between the *Cabp5* pattern and the average profile for nine genes expressed in differentiating rod photoreceptor cells (5.05 ± 0.37 normalized tag units; *P* = 0.0425 by Student's one-tailed t-test), suggesting that at least a subset of bipolar cell-enriched genes have temporal expression patterns that cluster in a distinct window apart from patterns for a subset of rod photoreceptor cell-enriched genes.

The late-expressed, previously characterized rod photoreceptor cell-enriched genes included rhodopsin (*Rho*; Molday and MacKenzie, 1983; Jan and Revel, 1974), guanylate cyclase activator 1a (*Guca1a*; Subbaraya et al., 1994), aryl hydrocarbon receptor-interacting protein-like 1 (*Aipl1*; van der Spuy et al., 2002), G protein α1 (*Gnat1*; Lerea et al., 1986), rod outer segment membrane protein 1 (*Rom1*; Bascom et al., 1992), G protein γ1 (*Gngt1*; Peng et al., 1992), guanylate cyclase activator 1b (*Guca1b*; Howes et al., 1998), G protein-coupled receptor kinase 1 (*Grk1*; Zhao et al., 1998), and cGMP-specific phosphodiesterase 6G (*Pde6g*; Tuteja and Farber, 1988). These three additional candidate bipolar cell genes identified from SAGE libraries were demonstrated to be expressed in a highly enriched manner in bipolar cells as assessed by RNA in situ hybridization (see further below). Other genes exhibited temporal expression pattern similar to the profile of *Cabp5*, but these genes were not expressed in an enriched manner in bipolar cells as assessed by RNA in situ hybridization and were not pursued further (data not shown).

### RNA in situ hybridization analysis

**Fig. 2:**
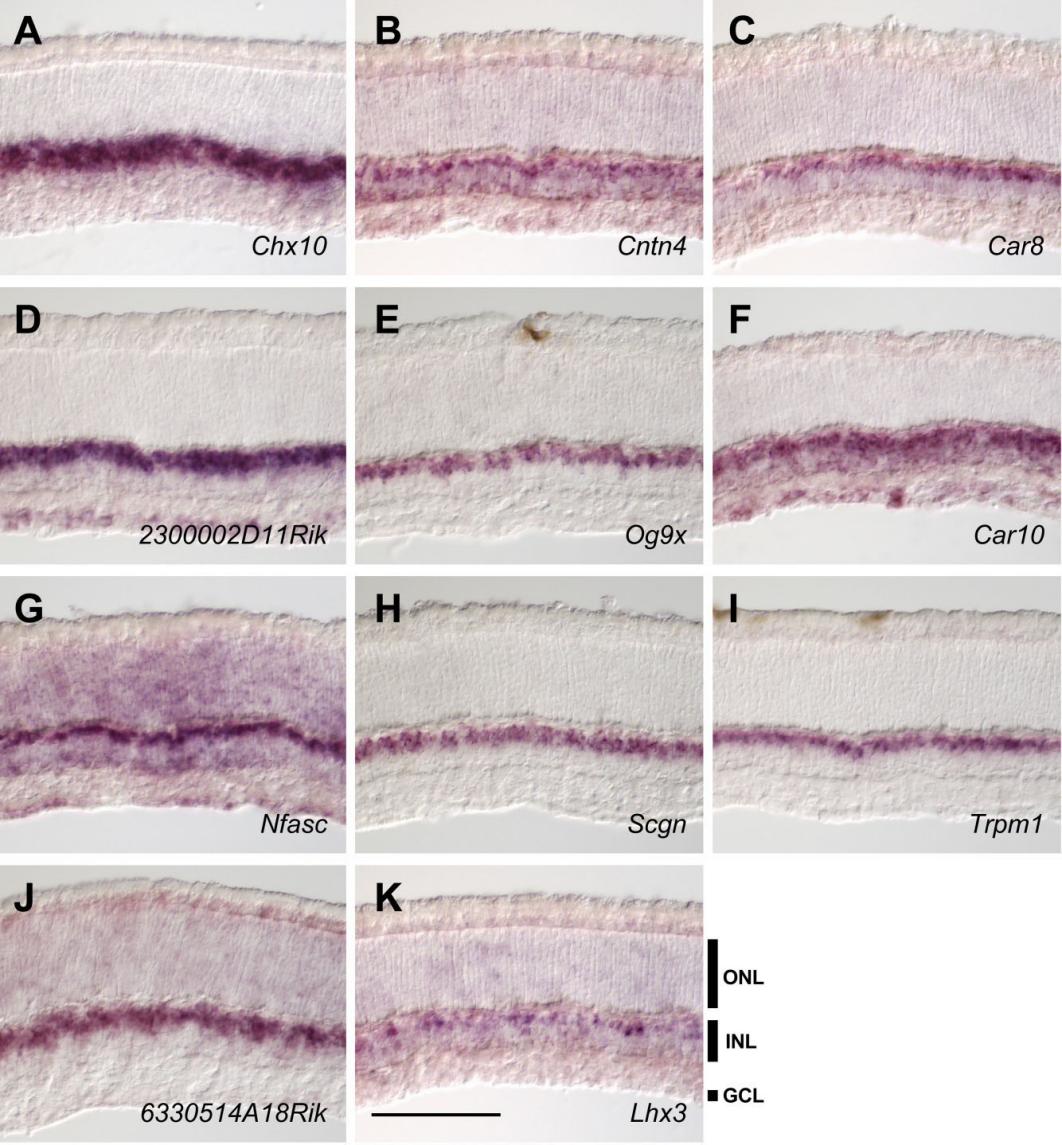
Expression patterns of novel bipolar cell-enriched gene candidates. RNA in situ hybridization patterns from representative sections of P21 mouse retinas. **A**: *Chx10*. **B**: *Cntn4*. **C**: Car8. **D**: *2300002D11Rik*. **E**: *Og9x*. **F**: *Car10*. **G**: *Nfasc*. **H**: *Scgn*. **I**: *Trpm1*. **J**: *6330514A18Rik*. **K**: *Lhx3*. ONL, outer nuclear layer; INL, inner nuclear layer; GCL, ganglion cell layer. Scale bar = 100 μm in K (applies to A–K).

RNA in situ hybridization analysis in P21 mouse retinal sections was carried out to evaluate expression of the candidate bipolar cell genes identified as described above. This validation process revealed that five of the candidate bipolar cell genes appeared to have highly enriched expression in bipolar cells, whereas five candidate bipolar cell genes exhibited enriched expression in bipolar cells and some expression in additional retinal cell types, as detailed below. For reference, the pattern for *Chx10*, a transcription factor gene expressed in all bipolar cells and a subset of Müller glial cells, is shown (Liu et al., 1994; Burmeister et al., 1996; Rowan and Cepko, 2004). *Chx10* expression was observed in cells on the outer (scleral) side of the INL where bipolar neuron cell bodies are located (Fig. 2A). Expression of the cell adhesion gene *Cntn4* was found in a subset of bipolar cells, when compared with the *Chx10* expression pattern, and weakly in a subset of amacrine cells, found in the inner (vitreal) part of the INL (Fig. 2B). *Car8* expression was seen in a subset of bipolar cells (Fig. 2C). The uncharacterized cDNA *2300002D11Rik* was expressed strongly in bipolar cells and weakly in the ganglion cell layer (Fig. 2D). The homeobox transcription factor gene *Og9x* was expressed in a subset of bipolar cells (Fig. 2E).

*Car10* expression was observed in a subset of bipolar cells and weakly in amacrine cells and the ganglion cell layer (Fig. 2F). Expression of the cell adhesion molecule *Nfasc* was found in a subset of bipolar cells and weakly in amacrine cells, photoreceptor cells, and the ganglion cell layer (Fig. 2G). The calcium-binding protein gene *Scgn* was expressed strongly in a subset of bipolar cells (Fig. 2H). *Trpm1* expression was seen in a subset of bipolar cells (Fig. 2I). The uncharacterized cDNA with homology to POLO-like kinase genes, *6330514A18Rik*, was expressed strongly in a subset of bipolar cells and weakly in the ONL (Fig. 2J). *Lhx3* was enriched in its expression eightfold in bipolar cells compared with rod photoreceptor and Müller glial cells in the microarray analysis, but the difference in expression levels was not significant (Table 2), perhaps because *Lhx3* is only expressed in a limited subset of bipolar cell types represented in the microarray data set. Consistent with this notion, expression of *Lhx3* was observed in a small subset of bipolar cells (Fig. 2K). Thus, the RNA in situ hybridization study results using retinal sections validated the utility of the microarray and SAGE screening. These screening methods were effective in identifying genes expressed in an enriched manner in bipolar cells.

**Fig. 3:**
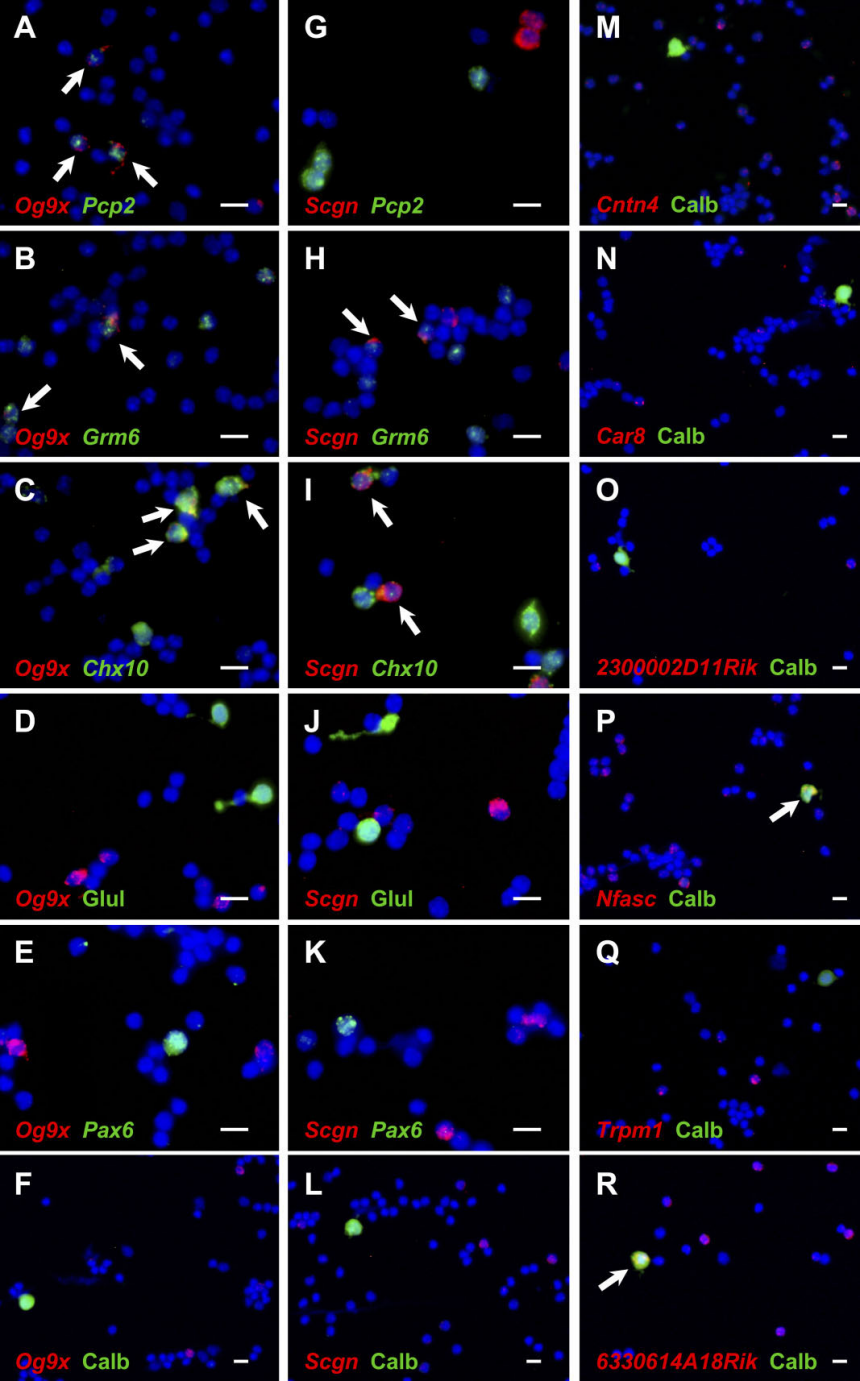
Characterization of novel bipolar cell-enriched genes by double-labeling with markers of bipolar cell subtypes and other retinal cells. Fluorescent RNA in situ hybridization and antibody staining images from representative fields of dissociated P14 mouse retinal cells. **A**–**F**: *Og9x* hybridization signal is shown in red. **G**–**L**: *Scgn* hybridization signal is shown in red. A,G: *Pcp2* hybridization signal is shown in green. B,H: *Grm6* signal is shown in green. C,I: *Chx10* signal is shown in green. D,J: Glul antibody staining signal is shown in green. E, K, *Pax6* signal is shown in green. **F**,**L**–**R**: Calb antibody staining signal is shown in green. M: *Cntn4* hybridization signal is shown in red. N: *Car8* hybridization signal is shown in red. O: *2300002D11Rik* hybridization signal is shown in red. P: *Nfasc* hybridization signal is shown in red. Q: *Trpm1* hybridization signal is shown in red. R: *6330514A18Rik* hybridization signal is shown in red. Arrows, double-positive cells. Nuclei were stained blue with DAPI. Scale bar = 10 μm in A–R.

To evaluate which types of bipolar cells express the newly identified genes, double-label expression studies were carried out by using fluorescent riboprobes hybridized to dissociated cells from P14 retinas. Probes for novel bipolar cell-enriched markers were labeled with a red fluorophore, and co-expression with a rod bipolar cell-enriched gene, *Pcp2* (Berrebi et al., 1991), an ON bipolar cell-specific gene, *Grm6* (Nakajima et al., 1993; Vardi and Morigiwa, 1997), or a pan-bipolar cell gene, *Chx10* (Liu et al., 1994; Burmeister et al., 1996), each labeled by a green fluorescent probe, was assessed. Examples of double labeling are shown in Figure 3, and quantitation of the results by using computer-based image analysis is shown in Table 3. Bipolar cells are a relatively rare class of neurons in the mouse retina (∼10%) compared with rod photoreceptor cells (>70%; Young, 1985), and so the majority of dissociated cells were unlabeled by the known bipolar cell markers. Nonetheless, *Og9x*-positive cells were mostly also positive for *Pcp2*, *Grm6*, and *Chx10*, suggesting that *Og9x* is a rod bipolar cell-specific gene (Fig. 3A–C). The percentages of *Og9x*-positive cells that were also positive for *Pcp2*, *Grm6*, and *Chx10* were 69.8%, 83.7%, and 98.8%, respectively (Table 3; *Og9x* row; columns i, j, k). Rod bipolar cells are only a fraction of ON bipolar cells and bipolar cells in general (Euler and Wässle, 1995; Ueda et al., 1997), and consistent with the suggestion that *Og9x* is a rod bipolar cell-specific gene, only a fraction of *Grm6*-positive cells and *Chx10*-positive cells were also *Og9x* positive (Table 3; *Og9x* row; columns g, h).

**Table 3:** Quantitation of Dissociated P14 Mouse Retinal Cells Double-Labeled with Fluorescent Riboprobes for Novel and Known Bipolar Cell-Enriched Markers^1^

(a)	(b)	(c)	(d)	(e)	(f)	(g)	(h)	(i)	(j)	(k)
Experimental probe	Experimental	*Pcp2*	*Grm6*	*Chx10*	Doubly labeled with experimental and *Pcp2*/*Pcp2*	Doubly labeled with experimental and *Grm6*/*Grm6*	Doubly labeled with experimental and *Chx10*/*Chx10*	Doubly labeled with experimental and *Pcp2*/experimental	Doubly labeled with experimental and *Grm6*/experimental	Doubly labeled with experimental and *Chx10*/experimental
*Og9x*	5.6 ± 0.5	9.5 ± 2.1	9.9 ± 2.1	13.9 ± 1.6	54.4 ± 4.0	33.5 ± 4.8	34.3 ± 6.1	69.8 ± 6.2	83.7 ± 2.3	98.8 ± 0.6
*Scgn*	5.2 ± 0.7	9.5 ± 1.7	10.0 ± 1.9	13.7 ± 4.7	10.5 ± 3.1	19.4 ± 4.5	17.1 ± 5.9	13.4 ± 1.6	34.4 ± 5.7	67.1 ± 20.3
*Cntn4*	8.8 ± 2.4	6.4 ± 0.0	10.2 ± 5.2	14.0 ± 5.3	56.1 ± 14.5	33.3 ± 3.6	47.1 ± 7.2	44.7 ± 9.0	46.5 ± 13.9	59.2 ± 21.0
*Car8*	7.0 ± 2.7	9.5 ± 2.1	9.9 ± 3.1	14.0 ± 2.7	62.8 ± 20.9	29.4 ± 14.8	47.6 ± 10.4	70.3 ± 5.8	75.1 ± 19.6	97.2 ± 2.8
*2300002D11Rik*	7.7 ± 1.5	9.5 ± 2.3	9.9 ± 3.6	14.1 ± 3.7	42.6 ± 10.9	31.7 ± 6.4	48.3 ± 11.8	60.0 ± 9.0	71.7 ± 16.7	60.9 ± 18.8
*Nfasc*	8.0 ± 2.2	9.5 ± 2.3	10.5 ± 3.7	14.0 ± 4.4	29.4 ± 1.3	41.8 ± 1.7	33.9 ± 14.6	67.1 ± 15.5	33.6 ± 8.8	82.2 ± 9.5
*Trpm1*	11.6 ± 3.1	9.5 ± 2.0	10.0 ± 1.3	14.1 ± 1.1	25.0 ± 5.2	76.3 ± 10.1	37.2 ± 5.8	37.7 ± 4.4	38.8 ± 8.6	83.3 ± 2.8
*6330514A18Rik*	18.5 ± 5.7	7.5 ± 2.3	10.0 ± 3.0	13.5 ± 1.5	41.2 ± 18.8	58.7 ± 19.8	24.9 ± 8.1	17.8 ± 6.9	38.5 ± 20.8	46.5 ± 12.5

1 Percentages are shown as average ± SEM. Results from three retinas were independently quantitated for each experiment. Percentages of cells labeled with various experimental probes, a probe for the rod bipolar cell-enriched gene *Pcp2*, a probe for the ON bipolar cell gene *Grm6*, and a probe for the pan-bipolar cell gene *Chx10*, are shown. Columns b–e list the percentage of cells labeled with probes out of the total number of cells. Columns f–h list the percentages of cells doubly labeled with a probe for an experimental and a known gene out of the number of cells singly labeled with a probe for a known gene. These percentages are the fraction of cells positive for a known gene that are also positive for an experimental gene. Columns i–k list the percentages of cells doubly labeled with a probe for an experimental and a known gene out of the number of cells singly labeled with a probe for an experimental gene. These percentages are the fraction of cells positive for an experimental gene that are also positive for a known gene.

In contrast, *Scgn*-positive cells were almost all *Pcp2* negative, even though the majority was *Chx10* positive, suggesting that *Scgn* is expressed in cone bipolar cells (Fig. 3G,I; Table 3; *Scgn* row; columns i, k). *Scgn*-positive cells were both *Grm6* positive and *Grm6* negative (Fig. 3H; Table 3; *Scgn* row; column j), suggesting that *Scgn* is expressed in both ON and OFF cone bipolar cells. Data for *Car10* and *Lhx3* are not shown because the fluorescent in situ hybridization signals for these genes were too weak to detect in dissociated cells. *Cntn4* is expressed in bipolar cells and amacrine cells, and from the double-labeling experiments, it was found in at least some *Pcp2*-positive cells and *Grm6*-positive cells. The majority of *Car8*-positive cells were also positive for *Pcp2*, *Grm6*, and *Chx10*, suggesting that *Car8* is a rod bipolar cell-specific gene. *2300002D11Rik* is expressed in bipolar cells and the ganglion cell layer, and from the double-labeling studies, it was observed in at least some *Pcp2*-positive cells and *Grm6*-positive cells.

*Nfasc* is expressed in bipolar cells and the amacrine and the ganglion cell layer, and it was detected in dissociated cells in at least some *Pcp2*-positive cells and a small fraction of *Grm6*-positive cells. *Trpm1*-positive cells were almost all labeled with the *Chx10* probe, but only a minority of cells was positive for *Pcp2* or *Grm6*, suggesting that *Trpm1* is expressed in cone OFF bipolar cells and in a small number of cone ON bipolar cells and rod bipolar cells. *6330514A18Rik* is expressed in bipolar cells and photoreceptor cells, and from the double-labeling experiments, it was found in a small minority of *Pcp2*-positive cells and in at least some *Grm6*-positive cells. Thus, the novel bipolar cell-enriched genes were expressed in a variety of different subtypes of bipolar cells.

Mouse retinal bipolar cell bodies appear intermingled in sections in the INL with horizontal cell and Müller glial cell bodies. Double-labeling experiments in dissociated retinal cells were conducted by using probes against novel bipolar cell-enriched genes and antibodies against calbindin (Calb), a protein expressed abundantly in horizontal cells and weakly in a subset of amacrine cells (Hamano et al., 1990; Elshatory et al., 2007), and Glul, a Müller glial cell marker (Riepe and Norenburg, 1977), to determine whether the novel molecular markers are found in these cell types. Additionally, the possibility that novel bipolar cell-enriched genes are expressed in amacrine cells was addressed by double-labeling dissociated cells using a *Pax6* riboprobe, which marks amacrine, horizontal, and ganglion cells (de Melo et al., 2003). Cells positive for Calb expression were negative for *Og9x* (0/11), *Scgn* (0/15), *Cntn4* (0/15), *Car8* (0/15), *2300002D11Rik* (0/14), and *Trpm1* (0/16) expression (Fig. 3, Supplementary Fig. 1). However, *Nfasc*- and *6330514A18Rik*-positive cells overlapped with strongly labeled Calb-positive cells (15/16 and 6/6, respectively), suggesting that these two genes are also found in horizontal cells or amacrine cells. Horizontal cells are so rare in the retina (<1%; Jeon et al., 1998) that quantitation of single-labeling results was not carried out. Fields were selected for examination based on presence of a Calb-positive cell, and then double-labeling was assessed. Only a small minority of Glul-positive cells was positive with probes for *Og9x*, *Scgn*, *Cntn4*, *Car8*, *2300002D11Rik*, *Nfasc*, or *Trpm1*, suggesting that these genes are not found in Müller glial cells at high frequency (Fig. 3, Supplementary Fig. 1, Supplementary Table 1).

Approximately one-third of Glul-positive cells were labeled with the probe for *6300514A18Rik*, indicating that a moderate number of Müller glial cells express this gene. Only a small minority of *Pax6*-positive cells was positive with probes for *Og9x*, *Scgn*, *Car8*, *2300002D11Rik*, or *Trpm1*, suggesting that these genes are not found in amacrine, ganglion, or horizontal cells at high frequency (Fig. 3, Supplementary Fig. 1, Supplementary Table 2). Among *Pax6*-positive cells, ∼30–40% were labeled by probes for *Cntn4*, *Nfasc*, or *6330514A18Rik*, indicating that a moderate number of amacrine, ganglion, and/or horizontal cells express these genes.

### Bipolar cell gene expression in *Bhlhb4*^−/−^ retinas

**Fig. 4:**
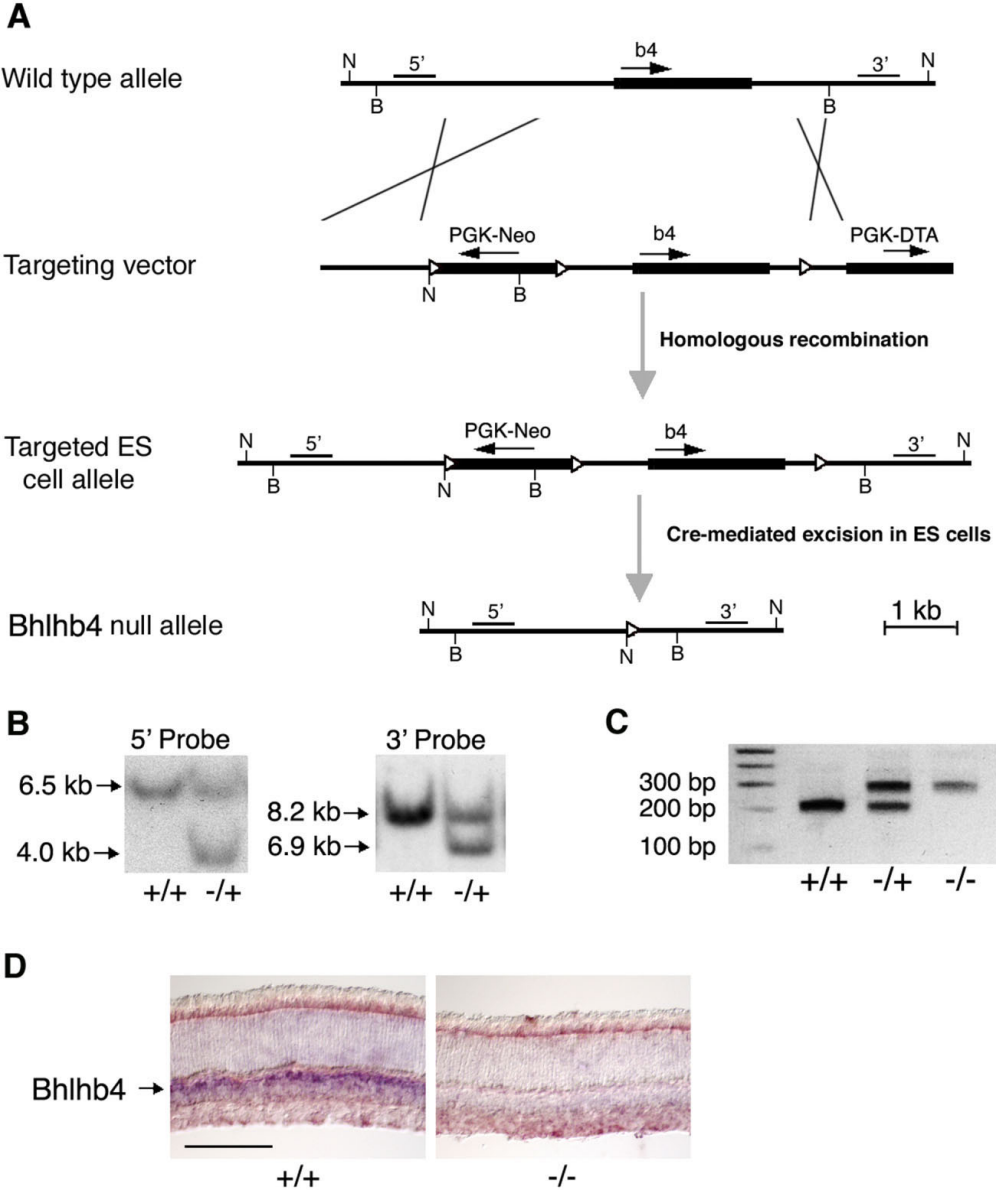
Mutagenesis of the mouse *Bhlhb4* gene. **A**: Gene targeting strategy showing partial restriction map of WT *Bhlhb4* allele, the targeting vector, the targeted ES cell allele, and the *Bhlhb4* null allele. The *Bhlhb4* gene (which is a single exon gene), the *PGK*-neomycin cassette, and the *PGK*-diptheria toxin cassette are represented by rectangles; the arrows represent open reading frames, and the triangles represent loxP sites. Thin lines show the positions of 5′ and 3′ probes used in Southern blotting analysis. *Bsm*I restriction sites (B), used for screening for integration by homologous recombination from the 5′ side of the gene, and *Nhe*I restriction sites (N), used for screening from the 3′ side, are indicated. **B**: Southern blot analysis of ES cells. Genomic DNA was digested with either *Bsm*I or *Nhe*I, and Southern blots were analyzed by using either the 5′ or the 3′ probe, respectively. Fragment sizes for WT (+/+) and targeted (−/+) DNA are indicated. **C**: PCR genotyping from mouse tail DNA from WT (+/+), heterozygous (−/+), and *Bhlhb4*-null (−/−) animals. WT allele, 216 bp; *Bhlhb4*-null allele, 299 bp. **D**: RNA in situ hybridization for *Bhlhb4* RNA. Retinal sections from P21 WT (+/+) and *Bhlhb4*-null (−/−) mice are shown. Scale bar = 100 μm in D.

**Fig. 5:**
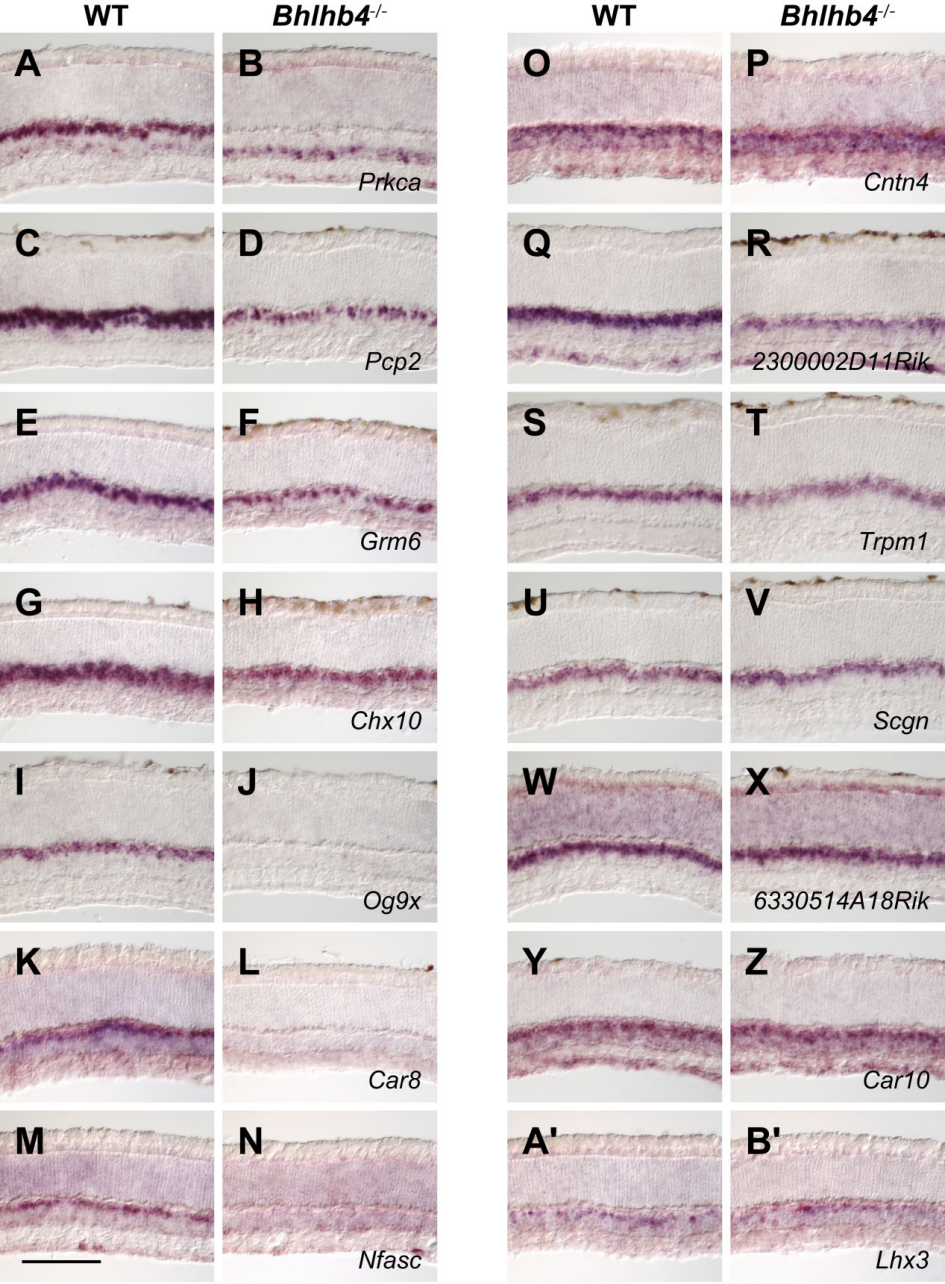
Bipolar cell gene expression in the *Bhlhb4*-deficient retina. RNA in situ hybridization patterns from representative sections of P21 mouse retinas. **A,C,E,G,I,K,M,O,Q,S,U,W,Y,A**′:, WT retinal sections. **B,D,F,H,J,L,N,P,R,T,V,X,Z,B**′:, *Bhlhb4*-deficient retinal sections. A,B: *Prkca*. C,D: *Pcp2*. E,F: *Grm6*. G,H: *Chx10*. I,J: *Og9x*. K,L: *Car8*. M,N: *Nfasc*. O,P: *Cntn4*. Q,R: *2300002D11Rik*. S,T: *Trpm1*. U,V: *Scgn*. W,X: *6330514A18Rik*. Y,Z: *Car10*. A′,B′: Lhx3. Scale bar = 100 μm in M (applies to A–Z,A′,B′).

To assess further the identity of bipolar cells in which the novel markers are expressed, in situ hybridization studies were conducted in retinas from P21 WT and *Bhlhb4*-deficient mice in which rod bipolar cells have previously been shown to die from P8 to P12 (Bramblett et al., 2004). A null mutation at the *Bhlhb4* gene locus was introduced by gene targeting and Cre/loxP-mediated recombination in ES cells, as shown in Figure 4. *Bhlhb4*-positive cells were completely absent in retinas of homozygous null mice as assessed by in situ hybridization (Fig. 4D). The specific loss of rod bipolar cells in *Bhlhb4* mutants was confirmed by examining *Prkca* expression, a known rod bipolar cell gene (Greferath et al., 1990). Whereas WT retinas displayed robust expression of *Prkca* in bipolar cell bodies closely apposed to the OPL and in a subset of amacrine cells, bipolar cells expressing *Prkca* were completely absent from the *Bhlhb4*-null retina; amacrine cell expression was maintained, however (Fig. 5A,B).

In the mutant retina, bipolar cells strongly expressing *Pcp2* and closely apposed to the OPL were lost, but weakly expressing bipolar cells located slightly closer to the center of the INL were retained, indicating that *Pcp2* is expressed in rod bipolar cells and a subset of cone bipolar cells (Fig. 5C,D). Fewer *Grm6*- and *Chx10*-expressing bipolar cells were evident in the mutant retina, reflecting the fact that these genes are expressed not only in rod bipolar cells but also in some cone bipolar cells, which remain (Fig. 5E–H). Similar analysis using the novel bipolar cell markers confirmed that *Og9x* (Fig. 5I,J) and *Car8* (Fig. 5K,L) are rod bipolar cell-specific genes because cells expressing these genes were absent from the mutant retina. Among bipolar cells, *Nfasc* also appears to be expressed specifically in a subset of rod bipolar cells (Fig. 5M,N). Slight to moderate reductions in bipolar cells expressing *Cntn4* (Fig. 5O,P), *2300002D11Rik* (Fig. 5Q,R), and *Trpm1* (Fig. 5S,T) were observed in mutant retinas, indicating that these genes are found in both rod bipolar cells and cone bipolar cells. *Scgn* expression was unchanged, consistent with the idea that *Scgn* is expressed in a subset of cone bipolar cells (Fig. 5U,V).

Additionally, bipolar cells expressing *6330514A18Rik* (Fig. 5W,X), *Car10* (Fig. 5Y,Z), and *Lhx3* (Fig. 5A′,B′) appeared unchanged in mutant retinas, indicating that these genes are expressed predominantly in cone bipolar cells. Supplementary Figure 2 shows the results of sense riboprobe hybridizations as negative controls. Table 4 lists the identities of the cells in which novel bipolar cell markers are expressed. Many of the known and novel bipolar cell molecular markers are also expressed outside the eye. These genes are represented in cDNA libraries from different tissue types in the Unigene database (http://www.ncbi.nlm.nih.gov/sites/entrez?db=unigene; Supplementary Table 3), and some genes are found to be expressed in various brain regions (Allen Brain Atlas, Lein et al., 2007; Supplementary Table 3).

**Table 4:** Summary of Expression of Novel Bipolar Cell-Enriched Markers

Gene name	Bipolar cell expression	Other retinal cell expression
*2300002D11Rik*	Rod bipolar cells, subset of cone ON and OFF bipolar cells	Ganglion cell layer
*6330514A18Rik*	Subset of cone ON and OFF bipolar cells	Photoreceptor cells, horizontal cells, Müller glial cells, amacrine cells
*Car8*	Rod bipolar cells	
*Car10*	Subset of cone bipolar cells	Amacrine cells, ganglion cell layer
*Cntn4*	Rod bipolar cells, subset of cone ON and OFF bipolar cells	Amacrine cells, ganglion cell layer
*Lhx3*	Subset of cone bipolar cells	
*Nfasc*	Subset of rod bipolar cells	Photoreceptor cells, horizontal cells, amacrine cells, ganglion cell layer
*Og9x*	Rod bipolar cells	
*Scgn*	Subset of cone ON and OFF bipolar cells	
*Trpm1*	Subset of rod bipolar cells, subset of cone ON and OFF bipolar cells	

## DISCUSSION

This study has revealed more of the complexity of gene expression of retinal bipolar cells and added a substantial number of bipolar cell-enriched molecular markers to those previously described. By using gene microarrays and SAGE data, genes were identified as candidates for novel bipolar cell molecular markers. RNA in situ hybridization studies showed the enriched expression to varying degrees in 10 genes in bipolar cells. These genes will prove useful in characterizing diverse bipolar cell types, understanding bipolar cell physiology, and elucidating bipolar cell development. This report complements studies that used genomic techniques to characterize retinal gene expression in general or to describe expression of a limited set of genes in bipolar cells (Huang et al., 2003; Blackshaw et al., 2004; Hackam et al., 2004; Dorrell et al., 2004; Zhang et al., 2005; Trimarchi et al., 2007).

The use of single bipolar cell cDNA as microarray probes and clustering of temporal expression patterns around that of a known bipolar cell gene by using SAGE data made a directed search for bipolar cell markers possible. In contrast to these methods, other investigators have identified genes fortuitously or by screening through molecules in families for which a member was already known to be expressed in bipolar cells. Another approach has been to find novel markers in the central nervous system (CNS) by undertaking comprehensive RNA in situ hybridization studies on a genome-wide scale (Lein et al., 2007). All these genomic techniques will prove generally useful in dissecting the complexity of gene expression in the nervous system.

In this study, not all previously identified bipolar cell markers were found. Notable missing genes were *Bhlhb4* and *Irx5*, transcription factor genes required for bipolar cell development or maintenance (Bramblett et al., 2004; Cheng et al, 2005), and *Gli5*, *Lhx4*, and *Zf*, bipolar cell molecular markers identified in a previous SAGE study (Blackshaw et al., 2004). Various factors underlie the relative sensitivity of the genomic screening approaches used here. These factors include lack of representation of some bipolar cell gene sequences on microarrays (e.g., *Grm6*), excessively low expression of bipolar cell genes on a cell-by-cell basis, temporal expression of bipolar cell genes outside the period of bipolar cell differentiation in which strongest SAGE clustering was found, and high expression of a given marker but only in a relatively rare bipolar cell type. With regard to this last factor, and most of the single bipolar cells examined in this study were picked based on expression of a transfected *Cabp5:GFP* reporter construct; *Cabp5* is only expressed in a subset of bipolar cells (rod bipolar cells, type 3, and type 5 cone bipolar cells; Ghosh et al., 2004). In retrospect, all these *Cabp5*-expressing cells appeared to be rod bipolar cells, based on high expression of the rod bipolar cell markers *Prkca* (Greferath et al., 1990) and *Og9x*. Consequently, many bipolar cell genes identified were rod bipolar cell-specific (e.g., *Og9x*, *Car8*) or at least were expressed in rod bipolar cells but were also found in cone bipolar cells (e.g., *Cntn4*, *2300002D11Rik*, *Trpm1*). More markers for cone bipolar cells, rather than rod bipolar cells, could be obtained by picking bipolar cells identified by using novel cone bipolar cell markers found in this study. Examining more single bipolar cells in general would also allow for quantitative comparisons of different cell types based on microarray signal intensities alone by increasing statistical power, but in this study, microarray data were used solely as a screening method and bipolar cell expression was ultimately evaluated by RNA in situ hybridization.

Evaluating expression of bipolar cell genes by RNA in situ hybridization was informative for understanding bipolar cell diversity. From double-labeling experiments, it was possible to estimate proportions of various bipolar cell types. For bipolar cells in general, the percentage of cells positive for the pan-bipolar cell marker *Chx10* was ∼14% (Table 3, column e), similar to other estimates of the bipolar cell percentage of 10–16% from comprehensive morphological studies (Young, 1985; Jeon et al., 1998). For ON bipolar cells, dividing the percentage of *Grm6*-positive cells (∼10%, Table 3, column d) by the percentage of *Chx10*-positive cells (∼14%, Table 3, column e), an estimate of the fraction of ON bipolar cells out of the total population of bipolar cells is obtained as ∼72%. Because *Chx10* is also expressed in a subset of Müller glial cells (Liu et al., 1994; Burmeister et al., 1996; Rowan and Cepko, 2004), the estimate of the fraction of bipolar cells out of the total population using *Chx10* is likely slightly overestimated, and the estimate of ON bipolar cells using *Grm6* and *Chx10* is likely underestimated. Previous studies using adult rat retina suggested that at least 50% of bipolar cells are rod bipolar cells based on antibody staining for protein kinase Cα and Purkinje cell protein 2 (Euler and Wässle, 1995). Based on the fraction of cells expressing the rod bipolar cell-specific markers *Og9x* and *Car8* out of the population of *Chx10*-positive cells, rod bipolar cells comprise ∼34–48% of all bipolar cells (Table 3, column h, *Og9x* and *Car8* rows). In this study, estimates were made by using P14 mouse retina. Differences in species, time points examined, and detection sensitivities could account for differences with previous figures. Some apoptosis of developing bipolar cells occurs in the mouse retina from P5 to P18, but the peak of death is evident at P8–P11, and only a few cells die thereafter (Young, 1984). Thus, the quantities of bipolar cells counted at P14 likely reflect the mature state. Additionally, *Nfasc* and *Trpm1* are expressed in a minority of cells expressing the rod bipolar cell-enriched gene *Pcp2*, which could reflect gene expression heterogeneity in these cells. Molecular differences among rod bipolar cells have not been explored and need to be confirmed by additional methods. Whereas this study has characterized overlap of gene transcripts with markers of different bipolar and INL cell subtypes, more sensitive methods for assessing gene or protein expression in the future could reveal that the novel markers are found in additional retinal cell types.

Expression in additional cell types does not necessarily exclude use of a marker. For example, *Chx10*, which is used as a pan-bipolar cell marker, is also expressed in Müller glial cells (Liu et al., 1994; Burmeister et al., 1996; Rowan and Cepko, 2004). In addition to the sensitivity of a detection method, other variables such as cell morphology can influence results. Double-labeling with the novel bipolar cell gene riboprobes and the Glul antibody showed expression of bipolar genes in a small percentage of Müller glial cells. These cells have long processes that can surround other cells even after dissociation, which can give a false impression of expression in Müller glial cells. Additionally, outside of the eye, cDNAs for novel and known bipolar cell-enriched molecular markers are represented in libraries made from several nervous system tissues, including brain, dorsal root ganglion, sympathetic ganglion, and spinal cord, as well as in non-nervous system tissues (see Supplementary Table 3). Many genes are also shown to be expressed in various brain regions when gene expression atlas data are examined (see Supplementary Table 3).

The expression studies using *Bhlhb4*-deficient retinas were revealing with regard to rod bipolar cells. Persistence of some *Pcp2*-positive cells in this mutant in which rod bipolar cells die and the rod bipolar cell component of the electroretinographic response is completely absent (Bramblett et al., 2004) suggests that *Pcp2* is expressed in some cone bipolar cells. Previous investigators concluded that Pcp2 is a rod bipolar cell marker, based on immunostaining of cells extending axons to the deepest part of the IPL (Berrebi et al., 1991). Visualization of *Pcp2*-positive cell bodies in the *Bhlhb4* mutant retina by in situ hybridization thus complemented immunohistochemical approaches investigating in which cell types this gene is expressed. It is possible that cone bipolar cells express *Pcp2* aberrantly as a consequence of rod bipolar cell loss. However, the remaining *Pcp2*-positive cells in the mutant retina were located relatively distant from the OPL and were expressed weakly. Similar *Pcp2*-positive cells were also seen in the WT retina, suggesting that *Pcp2* is normally expressed in a subset of cone bipolar cells. Given this indication that *Pcp2* is enriched in its expression in rod bipolar cells but is also found in a subset of cone bipolar cells, figures for the percentage likelihood that a novel marker is expressed in rod bipolar cells using *Pcp2* are likely underestimates. Furthermore, the analysis of the *Bhlhb4* mutants suggests that cone bipolar cell gene expression is unaffected by the absence of rod bipolar cells. Expression of the cone bipolar cell genes *Scgn* and *6330514A18Rik* was not changed in the mutant retina. In the absence of rod bipolar cells, the remaining cone bipolar cells appear unable to compensate for the loss, at least in terms of gene expression. Moreover, unlike cone photoreceptor cells, which undergo cell death after rod photoreceptor cells die in various forms of retinal degeneration, cone bipolar cells can persist even after rod bipolar cells are eliminated (Carter-Dawson et al., 1978; Bramblett et al., 2004). Understanding the causes of selective rod bipolar cell degeneration in *Bhlhb4*-deficient mice will require exploration of gene expression changes before and during the period of cell death (P8–P12). Bhlhb4 could regulate expression of trophic factor or trophic signal transduction genes that promote rod bipolar cell survival specifically. Alternatively, it might modulate expression of genes involved in rod bipolar neuronal activity, and rod bipolar cells could die as a consequence of abnormal physiological activity.

Exploration of the role of the novel bipolar cell genes could reveal more about bipolar cell development and function. *Cntn4* and *Nfasc* are both immunoglobulin domain-containing adhesion molecule genes (Volkmer et al., 1992; Yoshihara et al., 1995). Contactin protein family members participate in axon guidance and shaping dendritic projections, and *Nfasc* has been implicated in mediating specific neuron-neuron interactions (Falk et al., 2002). There is some evidence that contactin and neurofascin proteins can physically interact. Thus, it is possible that *Cntn4* and *Nfasc* function in the formation of bipolar cell axon structure and contacts. Additionally, the homeodomain-containing transcription factor genes *Og9x* and *Lhx3* are specifically expressed in bipolar cell subsets. Several homeodomain-containing transcription factors have been shown to be required for bipolar cell fate determination and differentiation (Burmeister et al., 1996; Chow et al., 2004; Ohtoshi et al., 2004; Cheng et al., 2005). The possibility that *Og9x* and *Lhx3* regulate bipolar cell development will be addressed in future gain- and loss-of-function experiments. Transcription factors that control bipolar cell gene expression and development could be investigated by examining common transcriptional regulatory elements of bipolar cell genes identified here and elsewhere, as has been done for photoreceptor cell genes (Qian et al., 2005; Hsiau et al., 2007). Thus, the identification of bipolar cell genes by genomic expression screening will reveal more about how development and diversification of these relay interneurons are regulated.
